# Supplementary material for: Investigating the use and impact of community Care (Education) and Treatment Reviews (C(E)TRs) in people with intellectual disability and autistic people: protocol for a cohort study using electronic health records
Source: BMJ Open. 2025 Sep 30;15(9):e107889. doi: 10.1136/bmjopen-2025-107889 (PMC12496089; doi:10.1136/bmjopen-2025-107889)
Supplement: online supplemental table 1 [file bmjopen-15-9-s001.docx]

**SUPPLEMENTARY TABLE 1**

**Baseline and time-varying socio-demographic and clinical variables**

| **Variable** | **Variable type** | **Variable description** | **Data source** |
| --- | --- | --- | --- |
| Age | Discrete | Age at index date (years) | Structured field |
| Sex | Categorical | Male, Female | Structured field |
| Ethnicity | Categorical | Asian, Black, White, Mixed, Other | Structured field |
| Sexual orientation | Binary | Heterosexual, Homosexual | Structured field |
| Religion | Categorical | Christianity, Islam, Hinduism, Other, No religious beliefs, Not Known | Structured field |
| Marital status | Categorical | Single, Married/Partnered, Not Known | Structured field |
| Who they live with | Categorical | Parent(s), Partner with/without children, Alone, Other, Not Known | Structured field |
| Deprivation score | Continuous | IMD (based on home postcode) | Structured field |
| Degree of intellectual disability | Categorical | Diagnosis; ICD-10 fields F70 (mild), F71 (moderate), F72 (severe), F73 (profound) | Structured field |
| Challenging behaviour | Binary | Yes/Not recorded; part of Intellectual Disability diagnosis; indicated by ICD-10 field F7-.1 | Structured field |
| Psychiatric diagnoses | Binary | Yes/No per diagnosis; categorized by ICD-10 subchapters | Structured field  and/or NLP |
| HoNOS | Count | Total score  Adjusted score (calculated based only on completed sub-scales)  Subscale scores  Variations of HoNOS may be collected depending on the data that are available (HoNOS, HoNOS-ID(LD), HoNOSCA, HoNOS65+) | Structured field |
| Hearing impairment | Binary | Yes/No | Structured field |
| Visual impairment | Binary | Yes/No | Structured field |
| Need for interpreter | Binary | Yes/No | Structured field |
| Previous in-patient admission | Binary | Yes/No; ever had an in-patient admission prior to index date | Structured field |
| Duration of previous in-patient admission | Count | Total number of days of admission prior to index date | Structured field |
| MHA section | Categorical | Which section of the MHA was used | Structured field |

HoNOS, Health of the Nation Outcome Scales; HoNOS-ID(LD), Health of the Nation Outcome Scales-Intellectual Disability (Learning Disability); HoNOSCA, Health of the Nation Outcome Scales Child and Adolescent; ICD-10, International Statistical Classification of Diseases, 10^th^ revision; IMD, Index of Multiple Deprivation; MHA, Mental Health Act; SLaM, South London and Maudsley

**SUPPLEMENTARY TABLE 2**

**6-month pre-/post-index date variables for sub-study 1 and sub-study 2**

| **Variable** | **Sub-variable** | **Variable Type** | **Variable description** | **CRIS/HES** | **Data Type** |
| --- | --- | --- | --- | --- | --- |
| Neuropsychiatric symptoms | Aggression | Binary | Yes/No based on any mention of the symptom | CRIS | NLP |
|  | Agitation |  |  |  |  |
|  | Anhedonia |  |  |  |  |
|  | Anxiety |  |  |  |  |
|  | Apathy |  |  |  |  |
|  | Arousal |  |  |  |  |
|  | Disturbed sleep |  |  |  |  |
|  | Emotional withdrawal |  |  |  |  |
|  | Fatigue |  |  |  |  |
|  | Hopelessness |  |  |  |  |
|  | Hostility |  |  |  |  |
|  | Insomnia |  |  |  |  |
|  | Irritability |  |  |  |  |
|  | Loneliness |  |  |  |  |
|  | Mood instability |  |  |  |  |
|  | Suicidal ideation |  |  |  |  |
|  | Violence |  |  |  |  |
| Neuropsychiatric symptoms scales | Positive schizophreniform | Count | Score [0 –16] | CRIS | NLP |
|  | Negative schizophreniform |  | Score [0-12] |  |  |
|  | Depressive |  | Score [0-21] |  |  |
|  | Manic |  | Score [0-8] |  |  |
|  | Disorganised |  | Score [0-8] |  |  |
|  | Catatonic |  | Score [0-4] |  |  |
| HoNOS |  | Count | Total score and subscale scores. Taken as HoNOS, HoNOS-ID(LD), HoNOSCA, or HoNOS65+ | CRIS | Structured field |
| Service Use - Admission to psychiatric hospital | Flag | Binary | Yes/No | CRIS | Structured field |
|  | Dates of admission / discharge | Dates | Start and end dates of hospital admission |  |  |
|  | Number of admissions | Count | Total number of admissions in window |  |  |
|  | Ward type on admission | Categorical | PICU, CAMHS ward, forensic ward, general psychiatric ward |  |  |
| Service Use - Care under the Home Treatment Team | Flag | Binary | Yes/No | CRIS | Structured field |
|  | Dates | Dates | Start and end dates of care |  |  |
|  | Days under the service | Count | Total number of days in window |  |  |
| Service Use – A&E Attendances |  | Count | Total number of admissions in window | HES | Structured field |
| Service Use -Admission to general hospital |  | Count | Number of attendances | HES | Structured field |
| Psychotropic medication |  | Binary | Yes/No | CRIS | NLP |

A&E, Accident and Emergency; CAMHS, Child and Adolescent Mental Health Service; CRIS, Clinical Record Interactive Search; HES, Hospital Episode Statistics; HoNOS, Health of the Nation Outcome Scales; NLP, Natural Language Processing; PICU, Psychiatric Intensive Care Unit
